# Use of free modified jejunal flaps to reconstruct pharyngoesophageal defects with preservation of the larynx in three cases

**DOI:** 10.1111/1759-7714.14469

**Published:** 2022-05-14

**Authors:** Jifeng Liu, Rong Yu, Ji Wang, Di Deng, Linke Li, Bo Li, Jun Liu, Fei Chen

**Affiliations:** ^1^ Department of Otolaryngology Head and Neck Surgery, West China Hospital Sichuan University Chengdu China

**Keywords:** cervical esophagus, free jejunum, hypopharyngeal cancer, laryngeal function preservation, swallowing

## Abstract

The larynx is often sacrificed in patients with hypopharyngoesophageal cancer before reconstruction using the jejunum to restore the continuity of the digestive tract and allow oral alimentation. We retrospectively collected and analyzed data from three patients who underwent hypopharyngoesophageal reconstruction by partial patch and partial tube free jejunal flap with preservation of laryngeal function. All three flaps survived in patients who underwent the modified jejunal flaps. The larynx was preserved in all three patients. Partial patch and partial tube jejunal flap is a possible option for reconstruction of large and complex defects after pharyngectomy and cervical esophagectomy with larynx preservation.

## Graphical abstract

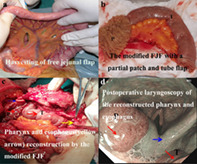

## INTRODUCTION

Revascularized free jejunum has been widely used to reconstruct the hypopharyngoesophageal tract.^1^ The larynx is sacrificed in most of those patients before they undergo reconstruction using the jejunum to restore the continuity of the digestive tract and allow oral alimentation. To fit the complex defect and preserve laryngeal function, we partially longitudinally incised the jejunum to perform partial patch and partial tube flap and reconstructed large and circumferential hypopharyngoesophageal defects in patients with hypopharyngeal squamous cell carcinoma (HSCC) with invasion of the cervical esophagus. This modified procedure might help to expand the indications for using the jejunum in pharyngoesophageal reconstruction with preservation of the larynx.

## CASE REPORT

Three male patients with advanced HSCC with invasion of the cervical esophagus who had no partial response to two cycles of induction chemotherapy (cisplatin +5‐fluorouracil) were enrolled in West China Hospital, Sichuan University. The preoperative clinical characteristics and stage of the three patients are listed in Table 1.

**TABLE 1:** Patient demographic data and postoperative information

Patients	Age(year)	Tumor range	TNM stage	Total parenteral alimentation time (days)	Oral feeding time (days)	Decannulation time (months)	Dysphagia	Reflux	Aspiration	Speech	Postoperative adjuvant treatment	Follow up (m)	Result
1	49	CE, PPW, unilateral PS	T_3_N_1_M_0_	3	21	4	No	No	Yes	Normal	Two cycles of DDP + 5‐FU + radiation (66Gy)	74	Lymph node metastasis of posterior pharyngeal space
2	53	CE, PPW, bilateral PS	T_3_N_2_M_0_	6	40	5	No	Yes	No	Normal	Radiation (60 Gy)	59	DSF
3	61	CE, unilateral PS, paraglottic space and arytenoid cartilage area	T_4_N_1_M_0_	3	21	4	No	No	No	Slightly worsened	Two cycles of DDP + 5‐FU+ radiation (50 Gy)	52	DSF

*Note*: The clinical staged according to International Union Against Cancer 2010.

Abbreviations: CE, cervical esophagus; DDP, cisplatin; DSF, disease‐free survival; PPW: posterior pharyngeal wall; PS, pyriform sinus.

**FIGURE 1:**
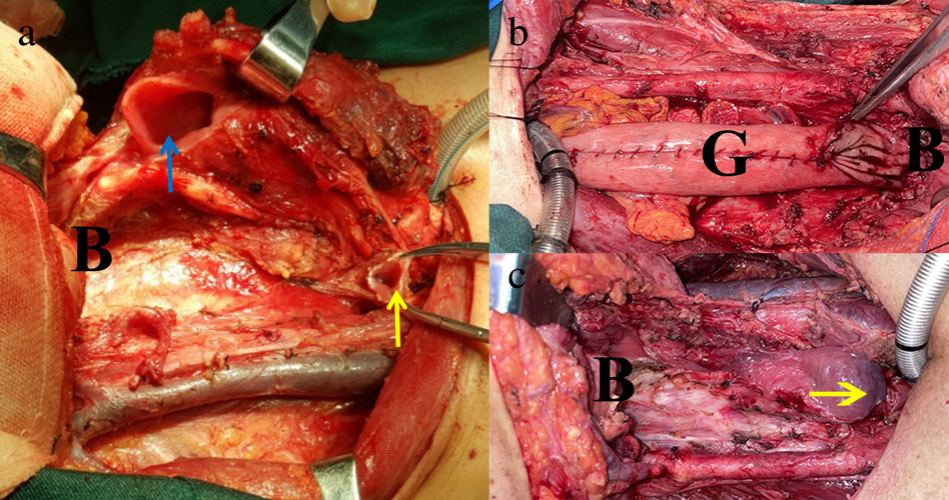
(a) The residual cervical esophagus (yellow arrow) and preserved larynx (blue arrow). (b) The gastric tube was used to repair pharyngeal and esophageal defects after total laryngopharyngectomy and cervical esophageal resection. (c) The defect after total laryngopharyngectomy and cervical esophagus resection. B, base of tongue; G, gastric tube

Bilateral neck selective dissection, cervical esophagectomy and tumor resection with a 1 cm safe margin were performed in the three patients (Figure 1a). Partial hypopharyngectomy was conducted in two of the three patients, total hypopharyngectomy was conducted in one of the three patients, and partial laryngectomy was conducted in one of the three patients. The larynx was partially or completely preserved in all patients. Meanwhile, possible available recipient vessels that could be used for microvascular anastomosis were carefully identified and preserved. During cervical esophageal resection, the recurrent laryngeal nerves were carefully dissected and protected. If possible, the superior laryngeal nerves were preserved.

**FIGURE 2:**
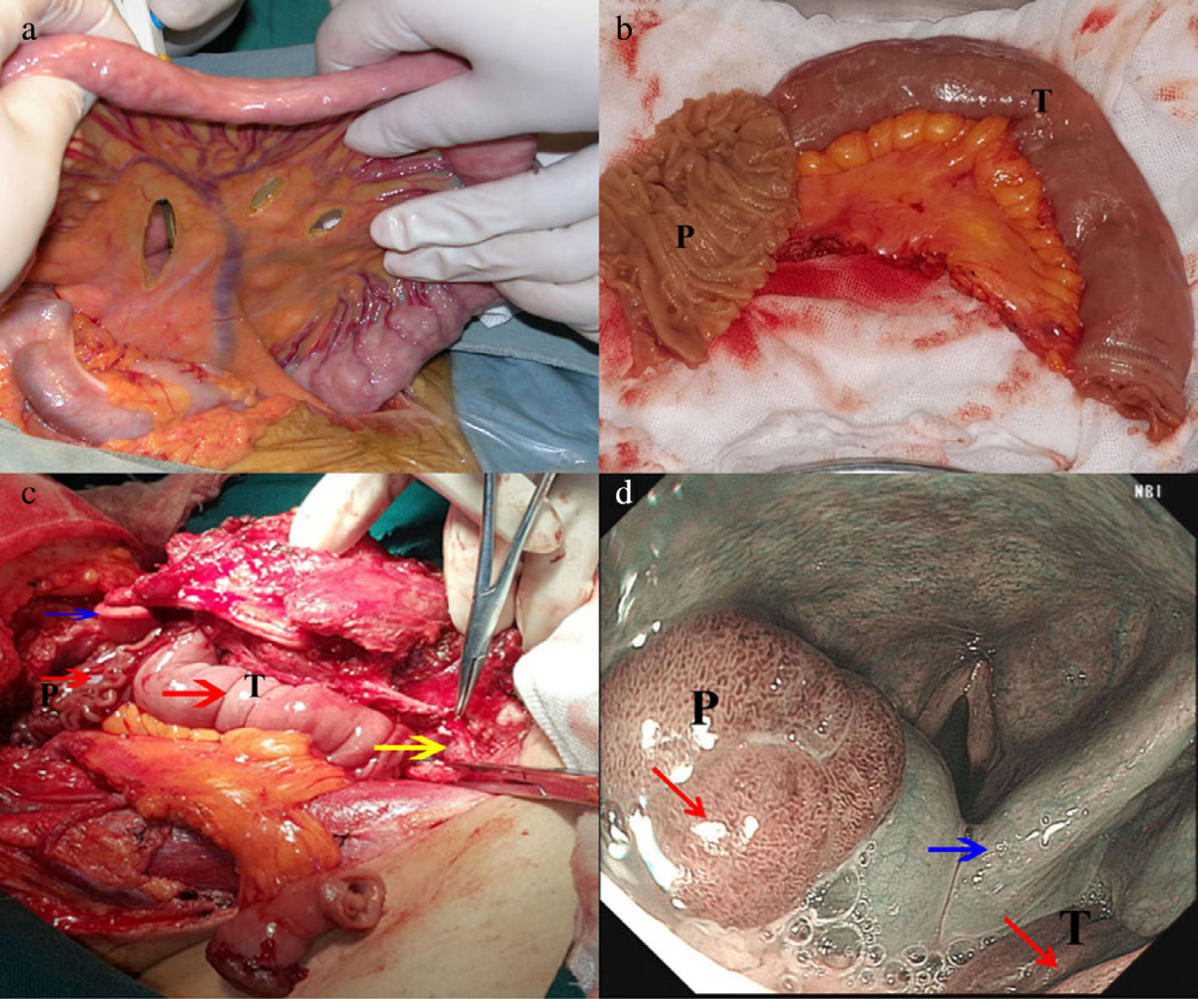
(a) Harvesting of free jejunal flap. (b) The free jejunal flaps were partially longitudinally dissected. (c) Pharynx and esophagus (yellow arrow) reconstruction. (d) Postoperative laryngoscopy of the reconstructed pharynx and esophagus. The red arrow shows the jejunal flap, and the blue arrow shows the larynx; P, partial patch‐free jejunal flap; T, tube‐free jejunal flap

A free jejunal flap (FJF) was harvested and partially incised in the longitudinal axis along the antimesenteric border to perform a free modified FJF with a partial patch and tube flap (Figure 2a,b). The free modified jejunal flaps were transferred to the defect site and sutured to the residual edge of the pharynx, esophagus and larynx (Figure 2c). The cervical esophageal defect was reconstructed with the jejunum tube, and a partial patch from the jejunum was used to repair the defect in the hypopharynx and larynx (Figure 2c,d). Vessel anastomosis was subsequently performed.

The operative complications and the function of the reconstructed pharynx, esophagus and larynx were evaluated. Additionally, the survival status of patients was recorded. Postoperative information is listed in Table 1.

No necrosis of the FJF was found in our three patients after surgery (Figure 2d). One patient had a subcutaneous infection. With treatment (e.g., drainage, dressing changes, systemic antibiotic treatment and nutritional support), the patient recovered within 4 weeks. None of the three patients had donor site complications.

After an average of 4 days of parenteral nutrition (range: 3 to 6 days), all of the patients began nasogastric feeding. The duration of oral feeding after the operation ranged from 21 to 40 days (first with semisolids and subsequently with a normal diet), and the length of the hospital stay ranged from 14 to 35 days. All three patients recovered swallowing function and were able to consume a normal diet after treatment. Aspiration was observed in one patient. However, the symptoms disappeared after 2 months of feeding train (from soft to liquid food). All patients were tracheostomy‐dependent after the operation, and the duration from surgery to decannulation ranged from 4 to 5 months.

After wound recovery, all patients received adjuvant radiotherapy, and two of the three patients also received chemotherapy (two cycles of cisplatin +5‐fluorouracil).

Two patients could communicate with others freely after treatment. Although one patient's speech function was affected by partial laryngectomy and he had a hoarse voice, he could still communicate with others by speech. The three patients had normal swallowing function.

The follow‐up time ranged from 52 to 74 months. Two patients had survived without tumor relapse and metastasis when the data were collected. One patient suffered from lymph node metastasis of the posterior pharyngeal space 36 months after treatment (Table 1). A second surgery followed by chemoradiotherapy was conducted for the patient. Interestingly, the FJF still functioned.

## DISCUSSION

Induction chemotherapy followed by radiotherapy or synchronous chemoradiation has been the main treatment for HSCC patients.^2^ In our patients, the tumors were not controlled by induction chemotherapy. For such patients, radical surgery usually includes total laryngopharyngectomy and cervical esophageal resection,^3^ which would lead to large and complex defects in the boundary between the respiratory system and the digestive tract (Figure 1b,c). In some challenging cases without laryngeal invasion, total laryngectomy is performed to facilitate defect reconstruction (Figure 1b,c).^4^ Among reconstruction procedures, the tube‐skin flap and gastric tube are not likely to fill large and complex defects because of their bulky volume and limited donor size (Figure 1b,c).^5^ In addition, the gastric pull‐up or stomach “patch” cannot be used to repair pharyngeal defects at higher positions because of the size of the stomach and the loss of stomach capacity.^5^

The jejunum is a reconstructive choice for hypopharyngeal and cervical esophageal defects. Miyamoto et al.^6^ used a jejunal patch to reconstruct a hypopharyngeal defect with laryngeal preservation. However, regardless of whether a jejunal tube or patch is used, it is still challenging for surgeons to repair complex hypopharyngeal defects with larynx preservation, especially when the cervical esophagus is invaded. To preserve the larynx, we partially incised the jejunum along the longitudinal axis to obtain partial patch and tube‐free modified jejunal flaps of an appropriate shape so that the FJF would be suitable for complex defects created by preservation of the larynx.

In our patients, all of the free modified FJF survived, even after radiation, which indicated that partial longitudinal incision of the free jejunum did not increase the risk of necrosis of FJF. Importantly, two patients with preservation of the larynx were tracheostomy‐independent and had normal speech function. Only one patient's speech function was affected. However, he could still communicate with others by speech and was not tracheostomy‐dependent. One patient suffered from posterior pharyngeal space lymph node metastasis after the first operation. The transplanted jejunum still survived after lymph node dissection followed by a second treatment with radical chemoradiotherapy.

Our study had some limitations. First, as shown in other studies, FJF has limitations, such as time consumption, risk of ischemia, congestion and high technical demands.^7^, ^8^ Second, only three patients were included in our study; therefore, additional subjects are needed to verify the value of this technique in future. Third, the functional evaluation of degluttory, respiratory and vocal functions requires objective tools.

## CONFLICT OF INTEREST

The authors declare that there are no conflicts of interest.
